# Jejunojejunostomy intussusception following conversion from gastric sleeve to gastric bypass: a case report

**DOI:** 10.1093/jscr/rjag379

**Published:** 2026-05-29

**Authors:** Katie Sandhovel, Laura Nereyda Plaza Grisanty, Monica Santos, Joseph Youssef

**Affiliations:** The Brooklyn Hospital Center, General Surgery Department, St. George’s University School of Medicine, Brooklyn, NY 11201, United States; The Brooklyn Hospital Center, General Surgery Department, St. George’s University School of Medicine, Brooklyn, NY 11201, United States; General Surgery Department, The Brooklyn Hospital Center, Brooklyn, NY 11201, United States; General Surgery Department, The Brooklyn Hospital Center, Brooklyn, NY 11201, United States

**Keywords:** intussusception, gastric bypass, bowel perforation, bariatric surgery complications, jejunojejunostomy

## Abstract

A 46-year-old woman presented 15 months after laparoscopic conversion of sleeve gastrectomy to Roux-en-Y gastric bypass with acute abdominal pain and lactic acidosis. Computed tomography demonstrated free intraperitoneal air and mesenteric swirl sign concerning for jejunojejunal intussusception. Emergent laparoscopy revealed retrograde intussusception at the jejunojejunostomy with three bowel perforations and feculent peritoneal contamination. After reduction, intraoperative assessment confirmed bowel viability with successful reperfusion, allowing primary two-layer repair of enterotomies rather than segmental resection. A Brolin stitch was placed to prevent recurrence, and the incidentally discovered Petersen space defect was closed with permanent suture. Despite developing postoperative septic shock requiring vasopressor support and intensive care, the patient recovered completely with preserved bowel length and normal function at follow-up. This case highlights the importance of prompt surgical intervention for post-bariatric intussusception and demonstrates that primary repair may be feasible when careful intraoperative assessment confirms bowel viability.

## Introduction

Jejunojejunal intussusception is a rare but potentially life-threatening complication following Roux-en-Y gastric bypass (RYGB), occurring in 0.4%–4.7% of patients [1]. This involves retrograde telescoping of the common channel into the jejunojejunal anastomosis, often leading to obstruction and potentially bowel ischemia and perforation [1, 2]. The pathogenesis is likely related to motility changes in the divided small bowel, mesenteric thinning following weight loss, or anastomotic staples acting as a lead point [2]. Standard treatment involves emergent surgical exploration with reduction and either enteropexy, anastomotic revision, or resection depending on bowel viability [1]. When perforation occurs, many surgeons advocate for segmental resection given concerns for ischemic injury. We present a case where prompt operative intervention with reduction, careful viability assessment, and primary enterotomy repair successfully avoided resection despite multiple perforations and feculent contamination.

## Case report

A 46-year-old woman presented with 1 day of worsening epigastric pain and nausea. Her surgical history included three cesarean sections, laparoscopic sleeve gastrectomy, and laparoscopic conversion to RYGB performed 15 months prior (August 2024). The RYGB was constructed in an antecolic configuration with stapled gastrojejunostomy and jejunojejunostomy anastomoses created using an EndoGIA stapler. She had lost 80 pounds since her initial bariatric procedure.

**Figure 1:**
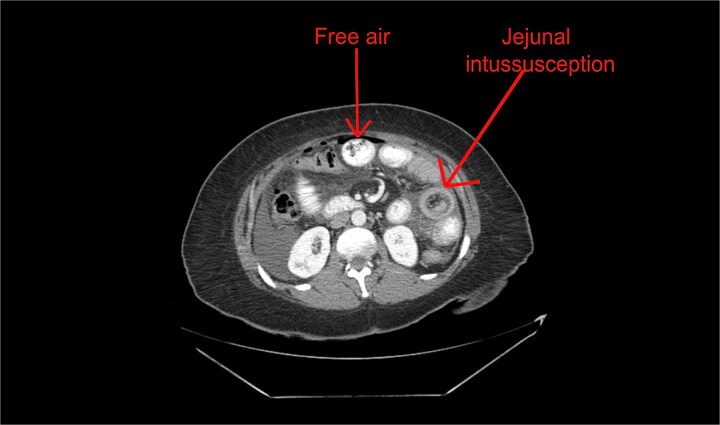
Contrast-enhanced CT demonstrating mesenteric swirl sign (arrow), venous congestion, and free intraperitoneal air consistent with bowel perforation.

Physical examination revealed abdominal tenderness. Laboratory studies demonstrated lactic acidosis (lactate 3.0 mmol/L) and acute kidney injury (creatinine 1.2 mg/dl). Contrast-enhanced computed tomography (CT) demonstrated free intraperitoneal air, mesenteric swirl sign, and venous congestion (Fig. 1), concerning for jejunojejunal intussusception with perforation. These findings prompted emergent surgical intervention.

### Operative findings and management

Emergent diagnostic laparoscopy was performed via four ports (one 5 mm left upper quadrant, one 5 mm midline, and two 5 mm right upper quadrant) with optical entry. Upon entering the peritoneal cavity, significant feculent and bilious succus was encountered, confirming bowel perforation with gross contamination. The abdomen was irrigated with 9 L of warm sterile saline for source control.

Inspection revealed retrograde jejunojejunal intussusception where the common channel had telescoped into the jejunojejunostomy (Fig. 4). The intussusception was reduced without difficulty. Upon reduction, three perforations were identified: two in the common channel and one at the jejunojejunostomy (Fig. 3).

**Figure 2:**
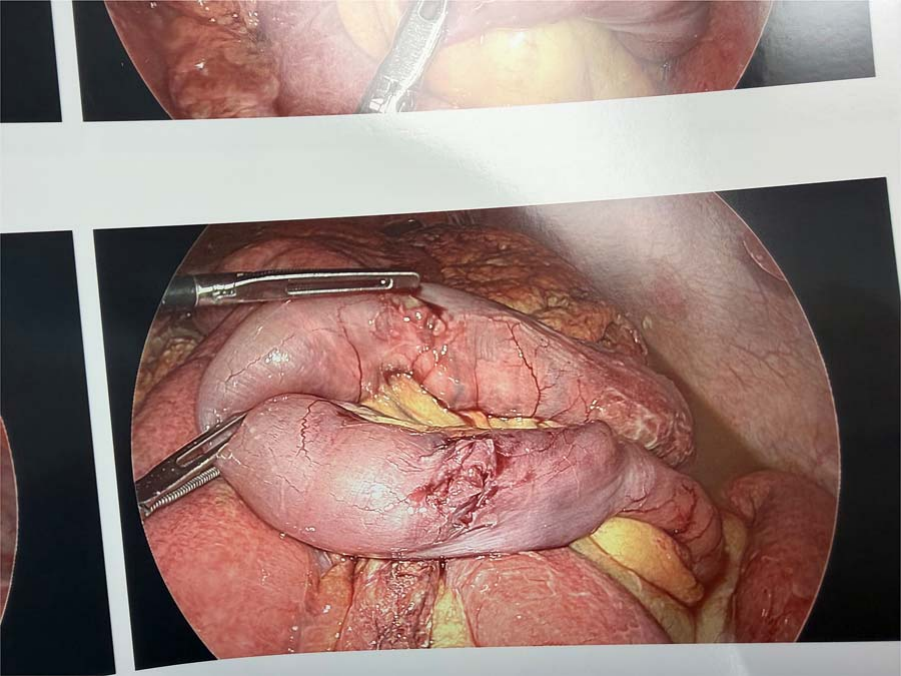
Laparoscopic view of bowel following reduction showing reperfusion with improved color and peristalsis, demonstrating viability without need for resection.

After reduction and irrigation, the bowel demonstrated successful reperfusion with return of normal color and peristalsis (Fig. 2). Given clear evidence of viability, the decision was made to perform primary repair rather than resection to preserve bowel length. The rationale included: prompt intervention before extensive ischemic injury, easy reduction suggesting an acute process, clear reperfusion after reduction, and ability to perform tension-free closure.

**Figure 3:**
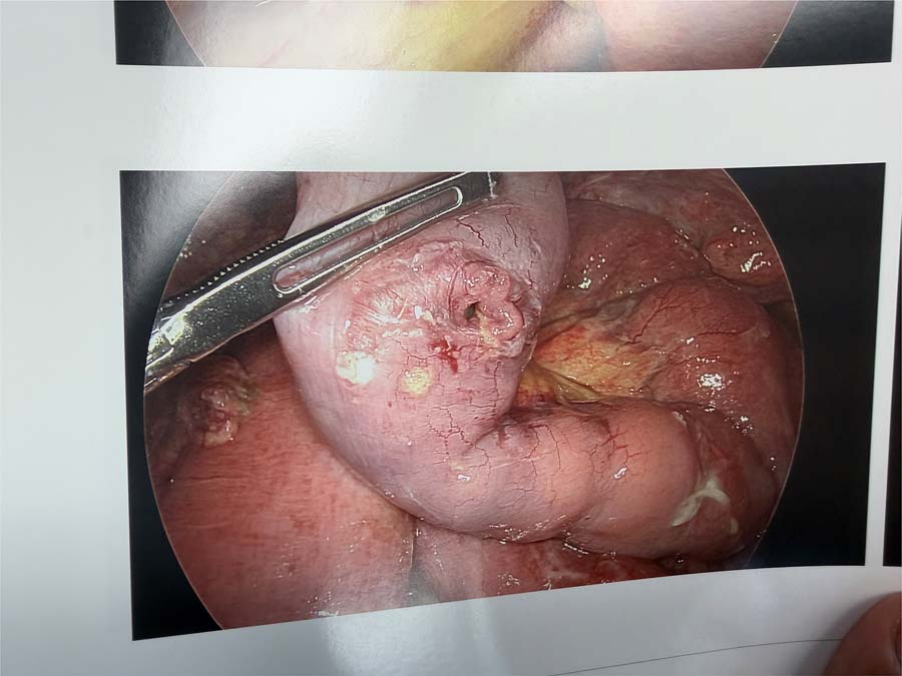
Laparoscopic image showing perforation in the intestinal wall discovered after reduction of the intussusception, demonstrating the ischemic injury caused by the intussusception.

**Figure 4:**
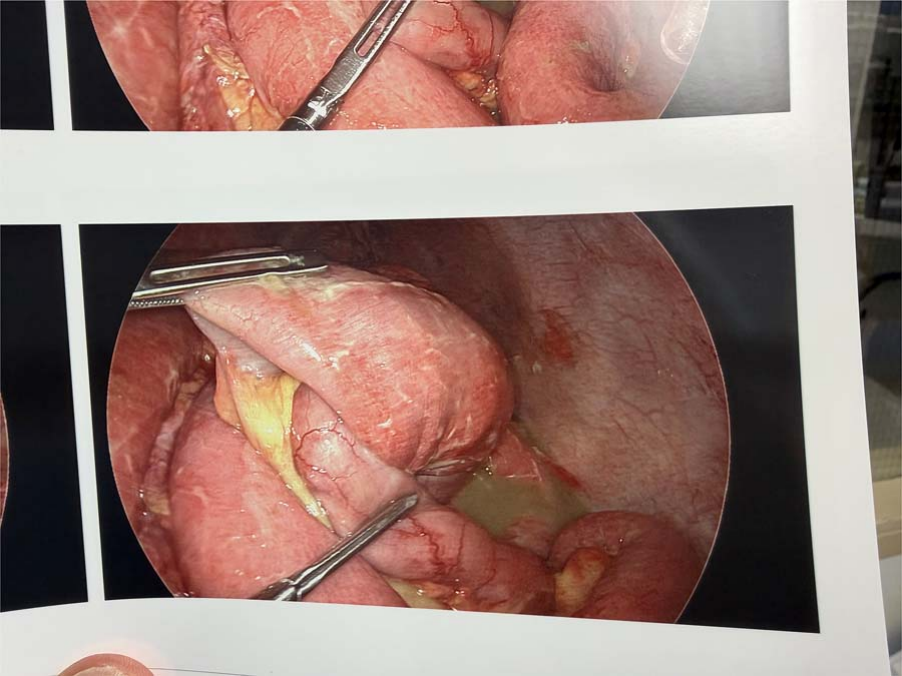
Laparoscopic view showing retrograde jejunojejunal intussusception with telescoping of the common channel limb into the jejunojejunal anastomosis.

The enterotomies were repaired using two-layer closure with 3–0 Vicryl sutures. A Petersen space defect was identified and closed with 2–0 Ethibond suture to prevent internal hernia. To prevent recurrent intussusception, a Brolin stitch was placed to anchor the common channel and biliopancreatic limb. Intraoperative esophagogastroduodenoscopy with leak test confirmed gastrojejunostomy integrity. Three Blake drains were placed near the gastrojejunostomy, jejunojejunostomy, and common channel repairs.

### Postoperative course

In the post-anesthesia care unit, the patient developed septic shock with hypotension (systolic 80s mmHg), tachycardia (120 s bpm), and worsening lactic acidosis (peak 7.0 mmol/L). Point-of-care ultrasound demonstrated a hyperdynamic left ventricle with collapsed inferior vena cava. She received aggressive fluid resuscitation totaling 8.5 liters of crystalloid and required norepinephrine infusion (maximum 10 mcg/kg/min). A femoral central line was placed for vascular access. She was admitted to the intensive care unit and treated with broad-spectrum intravenous antibiotics for intra-abdominal sepsis.

The patient was transferred to the surgical floor on postoperative day 4 after hemodynamic stabilization and vasopressor weaning. She demonstrated return of bowel function and was gradually advanced to a regular diet. Blake drains were removed prior to discharge. At follow-up on postoperative day 16, the patient reported normal eating and bowel function.

## Discussion

This case illustrates important principles in managing acute post-RYGB complications. First, it underscores the critical importance of prompt recognition and immediate surgical intervention when intussusception with perforation is suspected. The patient’s presentation with free air, lactic acidosis, and CT findings necessitated emergent exploration.

Second, this case demonstrates that intraoperative decision-making regarding resection requires careful assessment of bowel viability. While many surgeons advocate for segmental resection with perforation, primary repair may be feasible when reduction reveals viable bowel with successful reperfusion. Preserving bowel length was particularly important given the patient’s altered anatomy and potential for malabsorption.

However, this approach carried significant risk, as evidenced by postoperative septic shock. The feculent peritoneal contamination resulted in severe sepsis requiring intensive care. This underscores that primary repair in contaminated fields requires meticulous technique, adequate source control with copious lavage, appropriate drainage, and close postoperative monitoring.

Intussusception at the jejunojejunal anastomosis typically presents with abdominal pain, nausea, and vomiting, and can cause obstruction with subsequent strangulation and bowel necrosis if not recognized and treated promptly [1, 2]. The patient’s significant weight loss of 80 pounds over 15 months was a recognized risk factor, as rapid weight loss creates thinning of the mesentery that can predispose to intussusception [2]. Diagnosis is usually made with CT imaging, which often demonstrates a classic target sign, possibly with associated bowel thickening and mesenteric edema [1].

Treatment options for intussusception after gastric bypass vary from reduction with enteropexy to resection and reconstruction of the jejunojejunostomy [1, 3]. A systematic review found that resection of the affected segment was performed in 34% of patients, with a pooled recurrence rate of 22% during follow-up^4^. Resection and reconstruction of the jejunojejunostomy appears to be associated with the lowest risk of recurrence and acceptable complication rates [3]. However, in this case, the Brolin stitch placement represents an alternative approach to preventing recurrence while avoiding resection morbidity.

Closure of the Petersen space defect was important, as studies show that adding a purse-string suture at mesenteric defects reduces internal hernia rates from 12.9% to as low as 1.05% [4]. Internal hernia occurs in 3%–14% of patients after gastric bypass and can lead to similar complications if left untreated [5]. While incidental, closure was appropriate to prevent future complications.

## Conclusion

This case demonstrates that in carefully selected patients with jejunojejunal intussusception and bowel perforation following RYGB, prompt surgical intervention with reduction and primary enterotomy repair may preserve bowel length when intraoperative assessment confirms adequate viability. This approach requires two-layer closure, copious peritoneal lavage, adequate drainage, measures to prevent recurrence, and close postoperative monitoring. The development of septic shock underscores the substantial morbidity associated with bowel perforation in post-bariatric patients. Continued vigilance for intussusception in post-gastric bypass patients with significant weight loss remains essential given the risk of this rare but potentially life-threatening complication.
